# Heavy metals health risk assessment for population via consumption of food crops and fruits in Owerri, South Eastern, Nigeria

**DOI:** 10.1186/1752-153X-6-77

**Published:** 2012-08-01

**Authors:** Orish Ebere Orisakwe, John Kanayochukwu Nduka, Cecilia Nwadiuto Amadi, Daniel Onyekachi Dike, Onyinyechi Bede

**Affiliations:** 1 Toxicology Unit, Clinical Pharmacy Faculty of Pharmacy University of PortHacourt Rivers State Nigeria, PortHacourt, Nigeria; 2 Environmental Chemistry and Toxicology Research Unit, Pure and Industrial Chemistry Department, Nnamdi Azikiwe University, Nnewi, Awka Anambra State, Nigeria; 3 Deartment of Medical Lab Science, Faculty of Science, Rivers State University of Science and Technology, PortHacourt, Rivers State, Nigeria

**Keywords:** Heavy metal, Food crops, Toxicity, Dietary intake, Risk assessment

## Abstract

**Background:**

This study assessed lead, cadmium, and nickel level in food crops, fruits and soil samples from Ohaji and Umuagwo and Owerri in South Eastern Nigeria and estimated the potential health risks of metals. Samples were washed, oven-dried at 70–80°C for 24 h and powdered. Samples were digested with perchloric acid and nitric acid. Metals were analysed with Unicam Atomic Absorption Spectrophotometer.

**Result:**

The concentration of Pb, Cd, and Ni in Ohaji exceeded the maximum allowable concentrations for agricultural soil as recommended by EU. Lead, Cd, and Ni in the food crops were highest in Oryza sativa, Glycine max, and Pentabacta microfila respectively. Highest levels of Pb, Cd, and Ni, in fruits were detected in *Canarium schweinfurthii*, *Citrus reticulata*, *Ananas comosus* respectively. The true lead and cadmium intake for the rice based meal were 3.53 and 0.034 g/kg respectively. Whereas the true intake of lead and cadmium for the cassava based meal were 19.42 and 0.049 g/kg respectively.

**Conclusion:**

Local food stuff commonly available in South Eastern Nigeria villages may contribute to the body burden of heavy metal. This is of public health importance.

## Background

Contamination of foods by heavy metals has become an inevitable challenge these days. Air, soil, and water pollution are contributing to the presence of harmful elements, such as cadmium, lead, and mercury in foodstuff. The occurrences of heavy metals-enriched ecosystem components, firstly, arise from rapid industrial growth, advances in agricultural chemicalization, or the urban activities of human beings. These agents have led to metal dispersion in the environment and, consequently, impaired health of the population by the ingestion of victuals contaminated by harmful elements [1].

Flooding from heavy downpour may lead to horizontal leaching from dump sites causing metal uptake by root of crops; the rest may find their way into open water bodies and the entire aquatic ecosystem. The entry into food chain of these metals leads to increased susceptibility and exposure to metal poisoning of the local population.

A number of serious health problems can develop as a result of excessive uptake of dietary heavy metals. Furthermore, the consumption of heavy metal-contaminated food can seriously deplete some essential nutrients in the body causing a decrease in immunological defenses, intrauterine growth retardation, impaired psycho- social behaviors, disabilities associated with malnutrition and a high prevalence of upper gastrointestinal cancer [2]. In Nigeria there is lack of food intake diaries to monitor the intake of heavy metals and therefore their levels in blood and urine. It is advocated that any legislation to check lead exposure to humans should be based on genuine scientific evaluation of the available data [3].

We have estimated the heavy metal (lead, cadmium, nickel and mercury) concentrations in soil and food crops commonly grown or sold in South Eastern Nigeria with the aim of evaluating the potential dietary toxicity by determination of daily intake of these metals. The effect of transfer factors of heavy metals from different soil sites are also studied in the food crops and fruits to quantify the concentration of accumulated metals to which the local population are exposed.

## Materials and methods

### Study area and sampling

Samples of some commonly grown fruits and food crops were collected from three different sites namely Ohaji (1, 2 & 3), Umuagwo and Owerri all in Imo State in southern Nigeria. For metal analysis, only the edible parts of food crops, and fruit samples were used.

### Sample preparation

All the collected samples of various food crops samples were washed with deionised to remove airborne pollutants. The edible parts of the samples were weighed and air-dried for a day, to reduce water content. All the samples were then oven-dried at 70–80°C for 24 h, to remove all moisture. Dried samples were powdered using a pestle and mortar and sieved through Muslin cloth.

### Digestion of the samples

For each food crop, three powdered samples from each soil site (0.5 g each) were accurately weighed and placed in crucibles, three replicates for each sample. The ash was digested with perchloric acid and nitric acid (1:4) solution. The samples were left to cool and made up to a final volume of 25 ml with deionised water. The hydrolysed samples were well shaken and transferred to a centrifuge tube for centrifugation at the rate of 3000 rpm to remove solid particles. The resulting homogenised samples were thoroughly mixed before sub-samples were taken for analysis to ensure homogeneity of the mixture. The presence of lead, cadmium and nickel were analysed in samples using the Unicam Atomic Absorption Spectrophotometer (AAS) Model 929) at 217.0, 228.8 and 232.0 nm wavelength respectively . The limit of detection for lead, cadmium, and nickel were all 0.001 ppm with blank values reading as 0.00 ppm for all the metals in deionized water with electrical conductivity value of lower than 5 μS/cm. Samples were analysed in triplicates.

### Quality control

Appropriate quality procedures and precautions were carried out to assure the reliability of the results. Reagents used to calibrate the instrumentation were of analytical grades. A spike-and-recovery analysis was performed to assess the accuracy of the analytical techniques used. Post-analysed samples were spiked and homogenized with varying amounts of the standard solutions of the different metals. The spiked samples were then processed for the analysis by the dry ashing method. Quality control measures were taken to assess contamination and reliability of data. The coefficients of variation of replicate analysis were determined for precision of analysis; the variations were found to be less than 10%.

### Data analyses

The daily intake rate of metals (DIR) was calculated by the following equation:

(1) $$\text{DIR}=C_{\text{metal}}\times D_{\text{food intake}}/B_{\text{average weight}}$$

Where C_metal_ is Heavy metal concentration in plants (μg/g)

D_food intake_ is daily intake of vegetable (kg/person)

B_average weight_ is average body weight.

The average adult body weights were considered to be 55.9kg, while average daily vegetable intakes for adults is considered to be 0.345 kg/person/day, respectively [4,5].

The true intake using the arithmetic mean according to Parkhurst (1998) [6] was calculated by multiplying contaminant level i.e. (yearly intake of rice, cassava and fruits multiplied by heavy metal contaminant for each of the food items). In all the estimated or calculated levels of lead and cadmium in the food samples yearly averages as quoted by various workers were used [7,8].

## Results

Table 1 shows the lead, cadmium nickel and mercury levels (mg/kg) in commonly consumed food crops in Nigeria. Lead ranged from 0.00 – 61.17 mg/kg found in *Oryza sativa*. The range of various metals in food crops were 0.00–61.17, 0.00-0.24, and 0.00-3.13 mg/kg and for lead (Pb), cadmium (Cd), and nickel (Ni) respectively. The highest levels of Pb, Cd, and Ni in the food crops were detected in rice (*Oryza sativa*), soybean (*Glycine max*), and *Pentabacta microfila* respectively.

**Table 1:** Lead, cadmium nickel and mercury levels (mg/kg) in commonly consumed food crops

**Sample**	**Pb**	**Cd**	**Ni**
Cocoyam (*Colocasia esculenta*)	0.22	Nd	0.58
Plantain (*Musa paradisiaca*)	Nd	0.11	1.00
Cassava (*Manihot spp*)	0.33	0.10	0.30
Yam (*Diascoria rotundata*)	Nd	0.11	1.04
Breadfruit (*Artocarpus altilis*)	Nd	0.24	1.04
Popcorn (*Senna didymobotrya*)	0.22	Nd	0.31
Edible mushroom	0.22	0.10	0.49
Groundnut (*Arachis hypogea*)	0.23	Nd	0.99
*Pentabacta microfila*	Nd	Nd	2.31
*Irvingia wombolu*	0.80	Nd	3.13
*Vigna subterranean*	1.01	0.13	2.64
Beans (*Phaseolus vulgaris*)	0.22	0.22	2.65
Soybean (*Glycine max*)	0.46	0.24	1.72
Potato (*Ipomea batatas*)	Nd	Nd	1.16
(*Tetracarpidium conophorum*)	8.52	0.14	1.92
Rice (*Oryza sativa*)	61.17	Nd	Nd
Maize (*Zea mays*)	1.01	Nd	Nd
(*Brachystegia eurycoma*)	0.90	Nd	0.71
sorghum (*Sorghum bicolor*)	Nd	Nd	0.53
Mucuna	Nd	Nd	0.48
Melon (*Cucumia melo*)	Nd	0.13	0.49
Wheat (*Triticum spp*)	Nd	Nd	0.39
Millet (*Penicum Milliaceum*)	3.54	Nd	0.44
(*Detarium microcarpum*	0.22	0.13	1.21

Nd = Not detectable.

Table 2 shows the lead, cadmium and nickel levels (mg/kg) in fruits. The range of various metals in fruits was 0.00-4.23, 0.00-0.24,and 0.00-1.76 mg/kg and for Pb, Cd, and Ni respectively. The highest mean levels of Pb, Cd, and Ni, in the fruits were detected in *Canarium schweinfurthii*, tangerine *Citrus reticulata*, and pineapple *Ananas comosus* respectively. Taken together, about 36% of the sampled food crops, and fruits had non-detectable levels of lead, 49% had non-detectable levels of cadmium, and 15.7% had non-detectable levels of nickel. Forty-five percent of the sampled food crops violated the permissible limits of 0.01 mg/kg and 0mg/kg for lead as prescribed by WHO, EU and EPA respectively, 49% violated the limits for cadmium and 82% violated the limits for nickel.

**Table 2:** Lead, cadmium nickel and mercury levels (mg/kg) in fruits

**Sample**	**Pb**	**Cd**	**Ni**
Guava (*Psidium guajava)*	0.58	Nd	Nd
Bannana (*Musa spp)*	0.46	Nd	Nd
Apple (*Malus spp.)*	0.22	Nd	Nd
Bush butter/African pear (*Dacryodes edulis*)	0.22	0.17	Nd
Grape (*Citrus paradise)*	0.33	0.14	0.08
Orange (*Citrus sinensis)*	0.34	Nd	0.08
Pawpaw (*Carica papaya)*	Nd	Nd	0.26
Avocado (*Persea Americana)*	Nd	Nd	0.72
Pineaple (*Ananas comosus)*	Nd	Nd	1.76
Local pear (*Canarium schweinfurthii*)	4.23	Nd	0.26
Tangerine (*Citrus reticulate)*	0.69	0.24	Nd
Bush mango (*Irvingia gabonensis*	Nd	Nd	0.22

Nd = Not detectable.

Heavy metal concentrations (mg/kg) in soil samples are shown on Table 3. The range of various metals in the soil samples was 0.00–3.53, 0.00-0.18, and 0.26-1.56 for lead (Pb), cadmium (Cd), and nickel (Ni) respectively. The concentration of Pb, Cd, and Ni in Ohaji 1, 2 and 3 soil samples exceeded the maximum allowable concentrations for agricultural soil as recommended by EU but lower than Canadian human quality health soil quality guideline when compared with Table 4.

**Table 3:** Heavy metal concentrations (mg/kg) in soil samples

**Soil sampling site**	**Pb**	**Cd**	**Ni**
Ohaji 1	0.68	0.16	0.90
Ohaji 2	3.53	0.10	1.56
Umuagwo	Nd	0.18	0.89
Owerri	0.22	Nd	0.26
Ohaji 3	0.22	0.10	0.69

Nd = Not detectable.

**Table 4:** Guideline for safe limits of heavy metals

**Sample**	**Standard**	**Cd**	**Pb**	**Ni**
Soil, ug·g^-1^	Indian Standard Awashthi	3-6	250-500	75-150
	WHO/FAO, 2007	-	-	-
	European Union, 2002	3-0	300	75
Soil, mg·kg^-1^	Canadian human quality health soil quality guideline	14	140	
Plant, ug·g^-1^	Indian Standard Awashthi	1.5	2.5	1.5
	WHO/FAO, 2007	0.2	5.0	**-**
	Commission regulation (EU, 2006)	0.2	0.30	**-**
Leaf vegetables Tubers, cereals and fruits	European Union maximum levels in foods (mg·kg^-1^ wet weight)	0.20 ^a^	0.3b	
		0.050 ^a^		
Stem vegetables, root vegetables, and potatoes		0.10 ^a^		
Bran, germ, wheat, and rice, Soyabean				

^a^ COMMISSION REGULATION (EU) No 420/2011 amending Regulation (EC) No 1881/2006 setting maximum levels for certain contaminants in foodstuffs.

^b^ FAO/WHO (2001), Joint Codex Alimentarius Commission.

Tables 5 and 6 show the daily intake rate (g person^-1^day^-1^) of lead, cadmium, nickel and mercury DIR through consumption of food crops, and fruits respectively. Rice (*Oryza sativa*) and Wall nut (*Tetracarpidium conophorum)* had the highest DIR of 0.3775 and 0.0526 for lead respectively. The highest DIR (0.0015) for cadmium were seen in Soybean (*Glycine max*) and Breadfruit (*Artocarpus altilis*), while the highest DIR for nickel were *Vigna subterranean* and Beans (*Phaseolus vulgaris*) with DIR of 0.0163 and Wall nut (*Tetracarpidium conophorum*) (0.0118).

**Table 5:** Daily intake rate (g person^-^¹day^-^¹) of heavy metals DIR through consumption of contaminated food crops

**Food crops**	**Pb**	**Cd**	**Ni**
Cocoyam (*Colocasia esculenta*)	0.0014	Nil	0.0036
Plantain (*Musa paradisiaca*)	Nil	0.0007	0.0062
Cassava (*Manihot spp*)	0.002	0.0006	0.0019
Yam (*Diascoria rotundata*)	Nil	0.0007	0.0064
Breadfruit (*Artocarpus altilis*)	Nil	0.0015	0.0064
Popcorn (*Senna didymobotrya)*	0.0014	Nil	0.0019
Edible mushroom	O.0014	0.0006	0.0030
Groundnut (*Arachis hypogea*)	0.0014	Nil	0.0061
Oil bean (*Pentabacta microfila*)	Nil	Nil	0.0142
*Irvingia wombolu*	0.0049	Nil	0.0193
*Vigna subterranean*	0.0062	0.0008	0.0163
Beans (*Phaseolus vulgaris*)	0.0014	0.0014	0.0163
Soybean (*Glycine max)*	0.0028	0.0015	0.0106
Potato (*Ipomea batatas*)	Nil	Nil	0.0072
Rice (*Oryza sativa)*	0.3775	Nil	Nil
Maize (*Zea mays)*	0.0062	Nil	Nil
*Brachystegia eurycoma*	0.0056	Nil	0.0044
Sorghum (*Sorghum bicolor*)	Nil	Nil	0.0033
Mucuna	Nil	Nil	0.0030
Melon (*Cucumia melo*)	Nil	0.0008	0.0030
Wheat (*Triticum spp*)	Nil	Nil	0.0024
Millet (*Penicum Milliaceum*)	0.0218	Nil	0.0027
*Detarium microcarpum*	0.0014	0.0008	0.0075

Nil = 0.

**Table 6:** **Daily intake rate (g person**^-^¹**day**^-^¹**) of heavy metals DIR through consumption of contaminated fruits**

**Fruits**	**Pb**	**Cd**	**Ni**
Guava (*Psidium guajava)*	0.0036	Nil	Nil
Bannana (*Musa spp)*	0.0028	Nil	Nil
Apple (*Malus spp.)*	0.0014	Nil	Nil
Bush butter/African pear (*Dacryodes edulis*)	0.0014	Nil	Nil
Grape (*Citrus paradise)*	0.0020	0.0009	0.0005
Orange (*Citrus sinensis)*	0.0021	Nil	0.0005
Pawpaw (*Carica papaya)*	Nil	Nil	0.0016
Avocado (*Persea Americana)*	Nil	Nil	0.0044
Pineaple (Ananas comosus*)*	Nil	Nil	0.0108
Local pear (*Canarium schweinfurthii*)	0.026	Nil	0.0016
Tangerine (*Citrus reticulate)*	0.0043	0.0015	Nil
Bush mango (*Irvingia gabonensis*	Nil	Nil	0.0014
Wall nut (Tetracarpidium conophorum)	0.0526	0.0009	0.0118

Nil = 0.

The estimated or the calculated intake of lead and cadmium according to Parkhurst equation is contained in Example of calculating true metal intake. The calculated amount of lead and cadmium for a person on rice, and tangerine (*Citrus reticulate)* and another person on Cassava (*Manihot spp*) and Bannana (*Musa spp).* The true lead and cadmium intake for the rice based meal were 3.531g/kg and 0.034 g/kg respectively. Whereas the true intake of lead and cadmium for the cassava based meal were 19.42 g/kg and 0.049 g/kg respectively.

### Example of calculating true metal intake

$\text{True Pb intake}=\left(24.8\text{ kg}/365\text{ days}\right)x\left(61.17\text{mg}/\text{kg}\right)+\left(200\text{ g x}0.69\text{ mg}/\text{kg}\right)=3.53g/\text{kg}$

$\text{True Cd intake}=\left(24.8\text{ kg}/365\text{days}\right)\text{x}0.0\text{ mg}/\text{kg })+\left(200\text{ g x }0.24\text{ mg}/\text{kg}\right)=0.034\text{ g}/\text{kg}$

$\text{True Ni intake}=\left(24.8\text{ kg}/365\text{days}\right)x\;0.0\text{mg}/\text{kg })+\left(200\text{ gx}0.00\text{ mg}/\text{kg}\right)=0.00g/\text{kg}$

$\text{True Pb intake}=\left(214\text{ kg}/365\text{days}\right)x0.33\text{mg}/\text{kg })+\left(200\text{gx}0.46\text{ mg}/\text{kg}\right)=19.42\text{ g}/\text{kg}$

$\text{True Cd intake}=\left(214\text{ kg}/365\text{days}\right)x0.10\text{mg}/\text{kg})+200\text{gx}0.0\text{ mg}/\text{kg})=0.049\text{ g}/\text{kg}$

$\text{True Ni intake}=\left(214\text{ kg}/365\text{days}\right)\text{x0.0.}30\text{mg}/\text{kg})+\left(200\text{gx}0.0\text{mg}/\text{kg}\right)=0.18g/\text{kg}$

i.e. (yearly intake of rice, cassava and fruits (tangerine and banana) multiplied by concentration of heavy metal contaminant in each of the food item).

## Discussion

The thrust of this study was to determine the levels of lead and cadmium, and nickel in commonly ingested farm produce that form the staple foods of Nigerians with a view to lending credence to the assertion by Gidlow (2004) [3] that irrespective of the pressure to reduce lead exposure in the general population and working environment, legislation must be based on genuine scientific evaluation of the available evidence. We have presented data on lead, cadmium, and nickel levels in food crops and fruits of southeastern Nigeria and the soil levels in corresponding farmlands. These values are higher in the sampled food crops and fruits compared to the soil samples. Some plants are capable of taking up lead from soil through their root systems, although this uptake does not appear to be appreciable [9] Soil samples from different farm lands where crops and fruits were harvested showed the presence of Pb, Cd and Ni. The concentration of soil lead was highest followed by Ni, and Cd in a descending order. The concentration of Pb, Cd, and Ni in Ohaji 1, 2 and 3 soil samples exceeded the maximum allowable concentrations for agricultural soil as recommended by EU but lower than Canadian human quality health soil quality guideline when compared with Table 4.

Cereals are a major component of the human diet and a source of essential nutrients, antioxidants and metabolites [10]. However, intake of toxic metal-contaminated cereals may pose a risk to human health. Agricultural activities have been identified as contributors to increasing toxic metal contamination through the application of various types of pesticides and fertilizers [11]. Results from present and previous studies [12,13] demonstrate that the foods grown on contaminated soils are more contaminated with heavy metals, which pose a major health concern.

In the present study the high percentage of food crops and fruits that violated the permissible limits of lead and cadmium as set by WHO, EU and EPA respectively is of public health concern. Among possible target organs of heavy metals, are soft tissues such as the kidney and liver and the central nervous system appear to be especially sensitive [14]. Some patients develop vesicular type of hand eczema following the ingestion of nickel in diet [15]. Although rare, chronic urticaria, a type 1 hypersensitivity response, has been attributed to dietary nickel [16]. The mean total dietary intake of nickel has been reported to be between 0.12-0.21 mg in UK [17], 0.13 mg in Finland [18], 0.17 mg in US [19] and between 0.207-0.406 mg in Canada [20]. The true intake of nickel in two Nigerian staple foods namely rice and cassava based meals calculated in this study appear to be within this range. This could be higher depending on food-fruits combinations. The high lead level seen in fruits especially local pear *Canarium schweinfurthii* is in an agreement with our previous finding of high lead level in same local pear in another city of South Eastern Nigeria [21].

Provisional tolerable weekly intake (PTWI) depends on the amount, consumption period and contamination level of consumed food. The FAO/WHO in 1993 established a provisional tolerable weekly intake (PTWI) of 25 μg lead/kg body weight for humans, equaling 1500 μg lead/week for a 60 kg person [22]. In 1995, the WHO estimated that total lead intake in adults worldwide range from 105 to 2212 μg/week [22]. In Canada the dietary intake of lead may be calculated to 168 μg/week for a 60 kg person [23]. The PTWI of cadmium has been set at 7 μg/kg body weight [24], equaling 420 μg cadmium/week for a 60-kg person. The dietary intake of lead, cadmium and nickel seem to be higher than the FAO/WHO PTWI.

Nigeria’s food regime is based essentially on two foods: grains, which provide 46% of calories and 52% of proteins consumed, and root crops/tubers, which provide 20% of calories and 8% of proteins consumed. Consumption of grains and root crops/tubers amounts to 150 kg and 214 kg respectively per person and per year [8]. The average Nigerian now consumes 24.8 kg of rice per year, representing 9% of total caloric intake [7]. The WHO/FAO report, "Diet, Nutrition and the Prevention of Chronic Diseases" recommends a population dietary intake goal of more than 400 g per day for fruits and vegetables. Many advanced counties have already launched campaigns for promoting the consumption of fruits and vegetables, especially in the framework of the International Fruits and Vegetables Alliance (IFAVA) [25,26]. In addition to the FAO-WHO initiative, such an approach is supported by the Global Horticultural Initiative (GlobalHort) and is now considered globally as a good way for reaching the United Nations Millenium Development Goals (MDGs) [26]. Should Nigeria adhere to this recommendation, it will be worthwhile to ascertain the safety of fruits consumption with respect to these heavy metals especially lead and cadmium. Although the present study has not employed the use of food intake diaries, the calculated/estimated intake of lead and cadmium using arithmetic mean, for a Nigerians on the popular staple foods namely rice or cassava were 3.53 - 19.42g/kg and 0.034 - 0.049 g/kg for lead and cadmium respectively. It could be feared therefore that if from staple foods alone excluding, the body burden of lead in an average Nigerian exceeds that of values obtained in Europe and America, a cumulative amount from other sources may make it even higher. Only recently Orisakwe 2009 [27] noted that while blood lead levels (BLLs) in many western countries have progressively declined over the years, in Nigeria high BLL continue to be documented not only in exposed workers but also in “unexposed” control subjects. There exist many sources of environmental lead exposure in Nigeria. At the top of the list is leaded gasoline. Although there was a plan to reduce the lead content of Nigerian gasoline from 0.74 g/L to 0.15 g/L by 2002 there is a doubt that it was implemented [28].

The degree of toxicity of heavy metals to human being depends upon their daily intake. Heavy metals intake through consumption of various types of food stuffs grown and sold in Southeastern Nigeria showed large variations. The standard of FAO/WHO (1999) [29] has established a reference value for tolerable daily intake. Our estimated daily intake rate for lead and cadmium DIR were above the tolerable daily intake rates for some of the food stuffs. In addition, the body weight of the human can influence the tolerance of pollutants. The DIM values for heavy metals were high when based on the consumption of food crops and fruits grown in the soils sampled in this study. The highest intakes of Pb, Cd, Ni and Hg were from the consumption of Rice (Oryza sativa), Soybean Soybean (*Glycine max*), oil bean (*Pentabacta microfila*) and (*Vigna subterranean*) respectively.

## Conclusion

Among other routes, food is one of the main sources of consumer exposure to heavy metals. Since increased dietary metals intake may contribute to the development of various disorders, there is a necessity for monitoring of these substances in the human diet. Heavy metals show a significant build-up with contamination and long-term accumulation of heavy metals in soils has led to contamination of food crops in the study area. All the food crops and fruits containing heavy metals were higher than the recommended tolerable levels proposed EU, USEPA and WHO. Local food stuff commonly available in South Eastern Nigeria villages may contribute to the body burden of heavy metal. This is of public health importance.

It is recommended that people living in this area should not eat large quantities of these foods, so as to avoid excessive accumulation of heavy metals in the body. Dietary intake of food results in long-term low level body accumulation of heavy metals and the detrimental impact becomes apparent only after several years of exposure. Thus regular monitoring of these toxic heavy metals from effluents and sewage, in foods is essential, to prevent their excessive build-up in the food chain.

## Competing interests

The authors declare that they have no competing interests.

## Authors’ contributions

OOE and NJK designed and handled write up, ACN analysed the data while DDO and OO handled collection of data, food sampling and laboratory experimentation. All authors read and approved the final manuscript.
